# Pulmonary Arterial Hypertension: A Deeper Evaluation of Genetic Risk in the -Omics Era

**DOI:** 10.3390/genes12111798

**Published:** 2021-11-16

**Authors:** Rajiv D. Machado, Laura Southgate

**Affiliations:** 1 Institute of Medical and Biomedical Education, St George’s University of London, London SW17 0RE, UK; rmachado@sgul.ac.uk; 2 Molecular and Clinical Sciences Research Institute, St George’s University of London, London SW17 0RE, UK

Pulmonary arterial hypertension (PAH) is a highly heterogeneous disorder with a complex, multifactorial aetiology. PAH is characterized by a sustained elevation of mean pulmonary arterial pressure resulting from the occlusion of distal pulmonary arterioles due to the uncontrolled proliferation of endothelial and smooth muscle cell populations. Of interest, pulmonary artery endothelial cells display monoclonal expansion suggestive of a neoplastic, quasi-cancerous phenotype. While the majority of PAH cases present with idiopathic disease (IPAH), approximately 10% of patients report a positive family history wherein the mode of transmission is predominantly autosomal dominant. More recently, examples of autosomal recessive inheritance have been recorded, albeit in rare instances. PAH may also be associated with co-morbidities, including congenital heart disease, connective tissue disease, thromboembolism and exposure to anorexigens (APAH). Of note, PAH displays a marked sex bias towards females (4.3:1) and reduced penetrance both within and between families. These features strongly suggest the presence of modifying factors that may be genetic and/or environmental in origin.

The identification of heterozygous, germline mutations in the bone morphogenetic protein receptor 2 (*BMPR2*) gene, encoding a type-II receptor of the TGF-β signalling superfamily, represented a critical breakthrough in defining the pathogenesis of PAH [1]. In the last decade, next-generation sequencing methodologies have been successfully harnessed in the detection of novel causative genes that, together, have significantly expanded the genetic landscape of this disease. However, several key clinical and molecular aspects of PAH remain enigmatic. First, missing heritability in both familial and idiopathic disease is yet to be fully explored and, indeed, solved. Historically, gene identification and variant analyses have largely targeted Caucasian, adult populations to the exclusion of paediatric cohorts and populations of different genetic ancestry that may provide important insights. A strong focus has been placed on the analysis of the exome in both gene identification and molecular diagnostic efforts. The comprehensive interrogation of non-coding regions of the genome may represent a fruitful means of accessing variation that explains the apparent mutation shortfall observed thus far. Modifying factors of PAH, for example, expression quantitative trait loci is a field that requires further investigation. Moreover, the preponderance of females afflicted with PAH remains a vexed question demanding deeper analysis. The cellular phenotype of PAH is considered to be largely similar to the Warburg phenomenon of cancer cell progression, namely monoclonal expansion of key cell populations, altered glycolysis and mitochondrial dysfunction [2]. However, this research avenue remains to be fully elucidated. Finally, although major breakthroughs have been made in the management and treatment of this devastating disease, clinical intervention remains ameliorative, and PAH typically continues to be a fatal outcome.

In this *Genes* Special Issue, a number of these outstanding questions are addressed. Welch et al. distinguish between the clinical and molecular genetic features of paediatric and adult PAH to draw distinct parallels between these disease subtypes [3]. Specifically, the authors indicate a significantly higher mutation burden in early onset disease (~42% by comparison to 12.5% in adults). Gelinas et al. performed whole-exome sequencing on a panel of paediatric patients to explore the genetic background to this specific and pernicious form of PAH [4]. This study provides novel insights into the wider mutation burden in childhood disease and also establishes the first independent validation of *BMP10* and *PDGFD* as genetic risk factors for PAH. Both reports emphasise the need to consider childhood-onset PAH as a genetically and clinically discrete entity to better facilitate improved diagnostic surveillance and novel gene identification.

The granular assessment of patient groups based on clearly defined ethnicity has furthered awareness of divergent genetic architecture in both PAH and other inherited diseases. To this end, Tenorio Castaño et al. surveyed a cohort of three hundred Spanish patients in a customised panel that included 21 genes associated with PAH [5]. Likely deleterious variants were detected in 15% of the patients analysed, including those with APAH. Moreover, the study recorded a sub-set of variants of uncertain significance (VUS) that require further annotation to reach clinical utility. The value of incorporating emerging genes, likely to underpin PAH, into existing screening protocols is exemplified by van den Heuvel et al., who added 17 newly identified causal factors to the previous diagnostic panel, which initially consisted of just *BMPR2* and *SMAD9*, in the Netherlands [6]. The diagnostic yield in a cohort comprising 28 previously tested patients and an additional 56 patients with IPAH or pulmonary veno-occlusive disease (PVOD) was subsequently augmented, thereby affording improved prospects for early diagnosis, screening of at-risk relatives and personalised management of patients. As detailed in some depth by Swietlik et al., genetic screening now plays a pivotal role in the diagnosis of PAH; yet, there is more work to be done to fully elucidate the genetic architecture, particularly in idiopathic disease. An intriguing approach is the assessment of stable intermediate phenotypes that, in turn, leads to a fluid interplay between forward and reverse genetics and reverse phenotyping. Whilst conceptual in nature, this study nonetheless suggests multi-omics approaches to otherwise intractable genetic disorders may yield subtle and/or masked insights into PAH pathogenesis [7].

With the exception of reports limited to single genes and small study panels, the non-coding regions of putative causative genes and the wider genome remain insufficiently investigated. An early example of the value of analysing these regions is provided by the identification of a deleterious variant in the 5′ untranslated region of the *BMPR2* gene, consolidated by functional evidence suggesting loss of transcript by nonsense-mediated decay [8]. To assess the role of non-coding sequence defects leading to the promotion of the disease phenotype, Song et al. examined nine different *BMPR2* promoter variants previously identified in seven PAH families and three IPAH patients by in vitro over-expression luciferase assays. In human pulmonary artery smooth muscle cells, the majority of these variants (c.-575A>T, c.-586dupT, c.-910C>T, c.-930_-928dupGGC, c.-933_-928dupGGCGGC, c.-930_-928delGGC and c.-1141C>T) significantly depressed activation of the reporter gene [9]. This preliminary examination of regulatory elements in known PAH risk factors draws much-needed attention to a hitherto poorly explored avenue of research into disease manifestation and modulation. Expansion of this proof-of-principle study into readily available whole-genome sequence data will establish a framework for the comprehensive determination of causal variation.

A powerful analytic technique for investigating the genetic triggers underlying PAH is driven by the assessment of expression quantitative trait loci (eQTL), a nexus between genetic association study signals and fundamental biology. Herein, Ulrich et al. describe transcriptome- and genome-wide eQTL mapping in an idiopathic and hereditary PAH cohort employing RNA sequencing (RNAseq) methodology [10]. By comparison to at least one published study, the authors confirm approximately 75% eQTLs from an identified total of 2314, of which 90% were cis-acting. These data implicate genes involved in immune-related processes, an acknowledged association of PAH pathogenesis. This insight has wider relevance to diseases that share a molecular signature with PAH.

Genomic instability in PAH has been a long-standing mechanism of the pathobiological process. To assess the importance of DNA repair in the nuclear and mitochondrial genome, Sharma et al. analysed aberration in damage response pathways in both the nuclear and mitochondrial genomes. Apoptotic escape associated with concomitant pro-proliferation processes provides a temporal sequence that may aid in early diagnosis and therapeutic intervention [11]. Of interest, expression profiles of key cellular sites of disease in PAH patients indicated that 586 genes were up-regulated and 372 down-regulated. Moreover, 35 of these genes were involved in DNA repair [12]. The authors report an intriguing link between *BRCA1* and BMP signalling, further providing an interface between PAH and neoplastic expansion.

Cirulis et al. posit the ‘oestrogen puzzle’ as a confounding factor in the development of PAH [13]. Typically, oestrogen and its metabolites protect organisms from developing indicative symptoms of pulmonary hypertension. However, in human disease, female carriers of causal variants are significantly more likely to develop the disease than male variant carriers. The authors in this review examine the interplay between oestrogen and the BMP signalling pathway in regard to the role of gender and PAH susceptibility. In relevant cell lines, the most prominent genetic risk factor, namely *BMPR2*, was significantly repressed by the administration of estradiol (E2) and estriol (E3). Indeed, over-expression in a cell line lacking native oestrogen receptors with elevated concentrations of ERα generated de-repression of the *BMPR2* promoter. Of interest, these findings have been reproduced in murine models; in particular *Bmpr2* gene expression was depressed in ovariectomized females. These studies indicate a multi-layered nexus between oestrogen signalling and BMP activity. The dysregulation of these pathways might promote a PAH phenotype that signals a shift toward mitogenic elements, for example, 16α-hydroxyestrone (16α-OHE_1_).

Treatment options in PAH have focussed on addressing pathobiological processes, namely endothelial dysfunction, excessive cell proliferation and vasoconstriction. Although efficacious in prolonging 5-year survival metrics, notably an improvement of 34% to 60%, these approaches are not curative, and the disease remains fatal often due to resistance to medication. Dannewitz Prosseda et al. propose employing the fundamental genetic and molecular findings key to disease pathogenesis as complementary avenues to the development of targeted, effective measures in combating this disease [14]. In particular, they note the fundamental importance of the BMPR2 pathway as a precision tool to the development of therapeutic modalities. Specifically, the authors present alternatives to current therapeutic strategies, which include enhancing BMPR2 availability at the cell membrane, relieving receptor inhibition, driving transcription of BMPR2 target genes or gene therapy approaches.

In conclusion, the series of articles presented in this Special Issue highlight that PAH genetics have now moved beyond a simple Mendelian ‘one gene = one disease’ model to the recognition of PAH as a multigenic and multifactorial disorder. Of note, independent validation of newly described genes by expert review has become an increasingly important consideration in the establishment of high-throughput diagnostic testing panels. By expanding contemporary analyses into considerations of the wider genomic architecture, these reports demonstrate that the examination of the non-coding genome, eQTL mapping, DNA damage repair and gender-specific bias will provide fundamental insights into the pathways and molecular mechanisms critical to the development and maintenance of the pulmonary vasculature. In the near future, further focussed investigations based on these findings will afford novel diagnostic and clinical avenues to improve outcomes for patients and their families.
